# Review of the History and Current Status of Cell-Transplant Approaches for the Management of Neuropathic Pain

**DOI:** 10.1155/2012/263972

**Published:** 2012-06-14

**Authors:** Mary J. Eaton, Yerko Berrocal, Stacey Q. Wolfe, Eva Widerström-Noga

**Affiliations:** ^1^Miami VA Health System Center, D806C, 1201 NW 16th Street, Miami, FL 33125, USA; ^2^Department of Cellular Biology and Pharmacology, Herbert Wertheim College of Medicine, Florida International University, Miami, FL 33199, USA; ^3^Department of Neurosurgery, Tripler Army Medical Center, 1 Jarrett White Road, Honolulu, HI 96859, USA; ^4^The Miami Project to Cure Paralysis, Miller School of Medicine at the University of Miami, Miami, FL 33136, USA

## Abstract

Treatment of sensory neuropathies, whether inherited or caused by trauma, the progress of diabetes, or other disease states, are among the most difficult problems in modern clinical practice. Cell therapy to release antinociceptive agents near the injured spinal cord would be the logical next step in the development of treatment modalities. But few clinical trials, especially for chronic pain, have tested the transplant of cells or a cell line to treat human disease. The history of the research and development of useful cell-transplant-based approaches offers an understanding of the advantages and problems associated with these technologies, but as an adjuvant or replacement for current pharmacological treatments, cell therapy is a likely near future clinical tool for improved health care.

## 1. Introduction

The transplantation of cells into the CNS for therapeutic purposes can be envisioned with increasingly demanding goals in mind: (1) local and sustained provision of therapeutic molecules, such as pharmacologic agents and neurotrophic factors; and (2) replacement of lost cellular populations and reconstruction of local neuronal circuitry. To a large extent, the demands of the particular therapeutic application will be the key factor in determining the goals of the transplant paradigm, and this guides both the selection of optimal cell type(s) and parameters (graft dose, site, timing, immunosuppressive regimen, etc.) for transplantation. With this in mind, the current review will explore the wide variety of approaches in neural transplantation that have been explored for the therapeutic management of pain. Since the goals of pain management can be widely disparate, approaches have evolved along distinctive paths during the progression of this field. Thus, while the provision of a local cellular source of biologic, rather than pharmacologic analgesic molecules may be appropriate in the management of some etiologies of new-developing neuropathic pain, cases of chronic, persistent neuropathic might require more heroic measures including replacement of lost neural populations and reestablishment of appropriate neurocircuitry. Nevertheless, some overlap in the goals of these requirements can be envisioned, for example, the provision of neurotrophic or neuroprotective molecules for the attenuation of hyperexcitability in acute pain or replacement of lost inhibitory neurocircuitry in chronic pain. Thus, this review will examine the history and development of the various cell types and paradigms, which run the gamut from primary tissue fragments to engineered stem cell lines, that have been taken in various models of chronic pain in order to identify breakthrough approaches in the treatment of these debilitating conditions.

During the past three decades, cell therapy as an approach to treat pain has progressed from a hypothesis for a method for modulating pain processing to the development of the first human cell sources that are being tested in clinical pain treatment. The near future will likely provide new challenges for the implementation in a wider audience of those who suffer chronic pain, considering problems common to all forms of cell transplantation, that is, immune rejection versus long-term survival and efficacy in the human host; dependable, well-characterized cell sources for grafts; cells that can safely integrate into or near the CNS, without danger of tumors or significant, deleterious effects; the ability to control the antinociceptive output of cell grafts, ideally increasing with the cyclic episodes of pain efficacy in a wide variety of pain causalities. However, cell therapy for pain offers much promise as a replacement or adjunct to current clinical methodologies, once the mechanisms of pain are well understood, so that such bioengineered cellular tools can be used appropriately. Although it is likely that the majority of the cell types grafted thus far are functioning much like a cellular minipump, providing neuroprotective and neurotrophic agents in the damaged CNS, more studies to implement lost neuronal circuitry to modulate pain need to be accomplished. Future studies will likely better define the processes and mechanisms that will lead to improved selection of cell types and trophic agents which can be utilized in combination to provide improved therapeutic outcomes following disease and injury to the nervous system that leads to debilitating pain.

## 2. Problem of Chronic Neuropathic Pain in Various Disease States and PNS/CNS Injuries

The treatment of neuropathic pain is perhaps one of the most difficult problems in modern clinical practice. In addition to affecting a large population, it is a staggeringly heterogenous diagnosis with multiple etiologies which respond in varied manners to a myriad of treatments. Current pharmacologic treatments often prove ineffective, must be used at impractical dose levels, or have unacceptable side effects.

While the prevalence of neuropathic pain had previously been conservatively estimated at 0.6% of the U.S. population [1], better definition, understanding and recognition of this diagnosis has led to a more realistic prevalence of 7-8%, recently reported in Europe [2, 3]. A recent review by the Neuropathic Pain Special Interest Group of the International Association for the Study of Pain (IASP) estimated the overall prevalence of neuropathic pain at 3.3 to 8.2% [4]. However, due to the large variation in reported prevalence and incidence, they also recommended further research efforts regarding the development of standardized methods for identification and assessment of neuropathic pain. Neuropathic pain usually presents with allodynia, pain felt in response to a normally innocuous stimulus, and hyperalgesia, increased sensation of suprathreshold nociceptive stimuli [5]. It is often associated with uncomfortable dysesthesias and may have continuous and paroxysmal components.

## 3. Etiologies

Neuropathic pain may result from disorders of the central nervous system, the peripheral nervous system, or may be mixed. Nearly any traumatic event or disease leading to neuronal damage, or neuropathy, has the potential to cause neuropathic pain. Causes of neuropathy include trauma, vascular and metabolic disorders, infections, neoplasms, toxins, autoimmune disease, as well as genetic and nutritional deficiencies. As the individual causes of neuropathic pain are seemingly innumerable, we will mention only the more common etiologies.

Trauma, usually in the form of spinal cord injury and also due to direct peripheral nerve damage, is one of the leading causes of severe neuropathic pain [6]. Traumatic neuropathic pain affects nearly 60% of individuals with spinal cord injury [5]. Neuropathic pain is most common at or below the level of injury and is often diffuse and poorly localized [7], making this type of pain difficult to deal with for the individuals with SCI [8]. Few treatments are effective and most individuals have to continue to leave with persistent pain [9]. While there are several pharmacologic agents that have shown success in clinical trials [10], the side effects are often ill tolerated at the dosages needed for relief.

One of the most common peripheral causes of neuropathic pain is diabetes. Diabetes mellitus is one of the most prevalent diseases in the United States, affecting 25.8 million (8.3% of the population) [11]. Nearly 70 percent of those with diabetes have diabetic peripheral neuropathy, which can lead to severe neuropathic pain of the distal extremities.

There are many infectious causes of peripheral neuropathy, including viral and bacterial etiologies. Viral infections are more common and usually result in more severe neuropathic pain. They include herpes varicella-zoster (shingles), Epstein-Barr virus, cytomegalovirus, and herpes simplex. The human immunodeficiency virus (HIV) can cause several different forms of neuropathy, including a rapidly progressive, painful polyneuropathy affecting the distal extremities which is often the first clinically apparent sign of HIV infection [12]. Lyme disease, diphtheria, and leprosy are bacterial diseases characterized by extensive peripheral nerve damage. Neoplasms can create neuropathic pain due to direct nerve compression and/or infiltration. While in a pure sensory nerve, these may be surgically resected; cases involving motor nerves or plexi are not amenable to surgical therapy and must be managed by pharmacologic or neuromodulatory treatments. Radiation, certain chemotherapeutic agents, and paraneoplastic syndromes can also result in peripheral neuropathy.

Toxins can result in a heterogeneous group of peripheral neuropathies. Those exposed to heavy metals, such as arsenic, lead, mercury, thallium, or industrial toxins, as well as certain therapeutic drugs in the anticonvulsant, chemotherapeutic, antiviral, and antibiotic classes, can also cause peripheral neuropathy and neuropathic pain. Nutritional deficiencies, in particular thiamine deficiency due to its prevalence amongst alcoholics, may also result in peripheral neuropathy. Trigeminal neuralgia is an excruciating cause of facial pain but is usually amenable to surgical vascular decompression of the nerve. Central neuropathic pain can be caused by multiple sclerosis and certain stroke syndromes [13]. Genetic and autoimmune neuropathies are rarer but add to the overall population of those with neuropathic pain.

## 4. Treatment

Given the widely diversified causes of central and peripheral neuropathy that can lead to neuropathic pain, it becomes much clearer as to the difficulty in treating this diagnosis. Clearly, the underlying condition is treated first, followed by symptomatic treatment of the pain. Unfortunately, only a dismal 40–60% of patients with neuropathic pain achieve partial relief. A significant roadblock of the treatment dilemma has been the difficulty with the most appropriate animal model to use for basic research, as well as the disconnect between behavioral outcomes examined in animal studies and those reported in the presentation of pain in a clinical setting [14].

In an effort to clarify the existing therapeutic paradigm for neuropathic pain, recent guidelines have been derived for pharmacological therapy [15–18]. These have combined evidence from randomized controlled trials with expert opinion and currently offer the clearest treatment paradigm. While certain drugs work best for specific indications, likely due to the mechanism of that disease etiology, it is becoming clearer that a combination of pharmacologics, adjuvant treatment and neuromodulation are usually needed to attain adequate pain relief [19–21].

Despite multiple therapeutic options, the treatment of neuropathic pain remains difficult and inconsistent. While combination therapy and an increasing number of adjunct therapies assist in treating intractable pain, there still remains no cure. Pain despite standard treatment due to poor efficacy, unacceptable side effects, and disease escalation demand continued investigation and development of new technologies to treat neuropathic pain.

## 5. Pain Classification

In the clinical setting, a broad classification of pain is made in order to differentiate between nociceptive and neuropathic pain types. This is a critical distinction because these pain types are dependent on partly different underlying mechanisms, and therefore, they usually require different treatment strategies. Arriving at an accurate neuropathic pain diagnosis is not straight forward, and this problem has been recognized both in pain research and in clinical pain management settings. To address this problem, the International Association for the Study of Pain (IASP) has proposed a modification of the present pain taxonomy. Neuropathic pain is currently defined as “pain initiated or caused by a primary lesion or dysfunction in the nervous system” [22]. However, Treede and colleagues [23, 24] recently suggested eliminating “dysfunction” from the definition and instead redefine the neuropathic pain definition to “pain arising as a direct consequence of a lesion or disease affecting the somatosensory system.” In order to standardize the classification into neuropathic and nonneuropathic pain, they recommended differentiating pain into “definite,” “probable,” and “possible” neuropathic pain. The following criteria were proposed to be required for “definite” neuropathic pain: (1) pain distribution consistent with injury to the peripheral nervous system (PNS) or the central nervous system (CNS); (2) history of an injury or disease affecting the PNS or CNS; (3) abnormal sensory signs within the body area corresponding to the injured area of the CNS or PNS; (4) a diagnostic test confirming a lesion or disease in these structures.

These criteria are applicable to many neuropathic pain conditions. However, in conditions with multiple concomitant pain types after CNS injuries, such as in SCI-related pain, distinguishing between neuropathic and nociceptive pain may be more difficult when pain is located in an area below the lesion but with partial sensory preservation. For such pain locations, abnormal sensory findings may not indicate a neuropathic pain diagnosis, since these will be present in the painful area regardless of whether pain is nociceptive or neuropathic [25].

Basic research studies have identified multiple underlying mechanisms of neuropathic pain and designed interventions to target them. However, significant knowledge gaps exist regarding the best methods to characterize pain subgroups (phenotypes) and their relationship to the underlying pain mechanisms in pain patients. A precise diagnosis is critical to the development of more effective treatments that are tailored to specific underlying mechanisms. Because neuropathic pain is dependent on multiple mechanisms [26], this knowledge gap is a significant barrier to translation of basic research finding into successful management of neuropathic pain.

The determination of clinical pain phenotypes is a promising way to classify pain types. This process may include statistical grouping of pain characteristics [27]. For example, a combination of the descriptive adjectives “burning,” “tingling,” “pricking,” “shooting,” and “freezing” pain, and evoked pain, differentiated between neuropathic and nonneuropathic pain in 618 patients with diabetic neuropathic pain, idiopathic neuropathic pain, or post-herpetic neuralgia or nociceptive osteoarthritis pain, or low back pain [28]. Another method used for defining clinical pain phenotypes includes quantitative sensory testing (QST; [29, 30]). Measurement of detection thresholds for tactile stimuli determines large-fiber and dorsal column-mediated function, and thermal detection and pain thresholds determine small-fiber and spinothalamic tract-mediated function. Thus, QST may facilitate the comparisons with basic research studies, since these studies often assess behavioral, evoked nociceptive responses.

## 6. Potential Strategies for Cell-Based Interventive Therapies: Rationale

### 6.1. Cellular Minipumps for Treatment of Pain

The earliest studies using cell transplants for pain were originally developed from the concept of descending inhibitory neurotransmitter modulation of sensory information [31], and that these same agents, such as catecholamines and opiates, released by cell grafts [32–36] after injury, could provide antinociception. Projections from midbrain, locus ceruleus, ventromedial, and ventrolateral medulla directly or indirectly terminate at spinal level to modulate incoming nociceptive signals. In addition, dorsal horn interneurons provide inhibitory influences at the same termini. A variety of neurotransmitters, peptides, opioids, and lately neurotrophins, such as BDNF, have been implicated in spinal inhibition. These include the endogenous neurotransmitters serotonin (5HT), noradrenaline, and gamma-aminobutyric acid (GABA); the endogenous opioids ß-endorphin, enkephalins, cannabinoids; endogenous peptides such galanin, and neurotrophins such as BDNF. Many of the commonly used pharmacologic therapies target these agents' receptors and reuptake mechanisms to increase or imitate their presence in acute and chronic pain. But it was recognized as early as 1980s [36] that these agents could be supplied by grafts of autologous adrenal medullary tissue [37] or chromaffin cells [38] which had been purified from adrenal tissue, and transplanted in a chronic arthritic pain model [39], after nerve transection [40], or peripheral nerve injury and painful neuropathy [41, 42] to attenuate behavioral hypersensitivity. Where a similar strategy using pharmacological agents and mechanical intrathecal delivery might be considered, intrathecal and/or intra administration cerebroventricular of opioids is limited by cost, the need for specialized maintenance and mechanical malfunctions if implantable drug delivery systems, or by the risk of bacterial contamination and ambulatory constraints when repeated daily injections via an intrathecal access port are used [43]. Intrathecal cell therapy secreting these same antinociceptive agents can be seen as an advantage. Since it was also noted that minimal immunosuppression was all that was required for good graft function and survival in the immune-protected nervous system [44], such studies led to the early initiation of clinical trials for chronic cancer pain with this cell transplant approach [42, 45, 46].

## 7. Early Use of Cell Therapy

### 7.1. Primary Adrenal Chromaffin Tissue and Cells

Some of the earliest studies utilized primary chromaffin cells in a rat model of neuropathic pain [41]. Chromaffin cells contain a cocktail of antinociceptive agents, peptides, and neurotrophins [47, 48]. These chromaffin cell grafts were placed either in midbrain structures [38], or in the lumbar subarachnoid space after partial chronic constriction injury (CCI) to the sciatic nerve [42], or after injection of formalin in the rat's hindpaw [49] for the antinociceptive effect. Many studies have sought to elucidate the agents released by these chromaffin grafts that might serve an antinociceptive role. These primary chromaffin cells grafts raise the levels of CSF met-enkephalin [32], increase CSF levels of catecholamines [50], and reduce morphine cross-tolerance [51] when used with morphine for pain. Changes in the spinal cord induced by nerve injury are attenuated by chromaffin grafts, such the induction of spinal NADPH-diaphorase [52] and cGMP [53], spinal c-fos induction [54], NMDA-induced hypersensitivity [55], and the loss of endogenous inhibitory GABA synthesis in the dorsal horn [56] that accompanies nerve injury. It is likely that adrenal transplants also block short-term spinal nociceptive facilitation, illustrated by the reduction in the c-fos induction by formalin in the presence of chromaffin grafts [57], probably by stimulating some persistent cellular process, such as increasing the descending inhibitory controls that regulate the firing of subpopulations of spinal cord nociresponsive neurons with release of opioids from grafted chromaffin cells, inhibitory modulation that may be an important determinant, but not the only one, of their analgesic effect [58]. To be able to use chromaffin cell therapy in humans, adrenal chromaffin cell grafts were prepared from xenogenic bovine sources and tested for antinociception after nerve injury [35, 59]. Such sources of primary bovine chromaffin cells have been safely used in initial trials with human patients with intractable cancer pain [60, 61]. But such primary tissue sources for the purification and use of chromaffin cells are not likely to be homogeneous, since they are often obtained from multiple donors. The ability to use and manipulate cell lines as a defined and stable source would be an alternate for eventual use in cell therapy.

**Figure 1:**
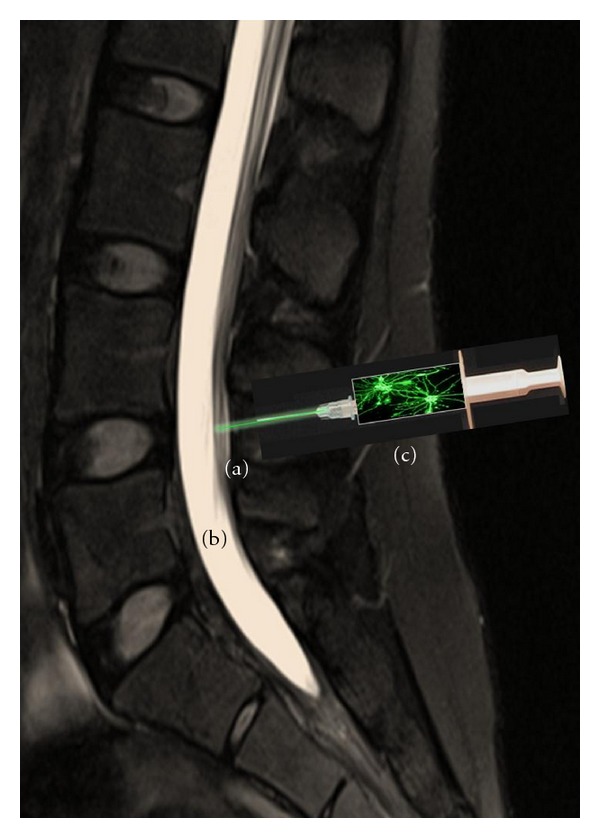
Model of lumbar subarachnoid injection of human neurons near the human spinal cord. MRI image of the human spinal cord (a) with a lumbar puncture of the subarachnoid space adjacent to the cord (b), and injection of cells, such as the GABAergic human neuronal hNT2.17 cells (c) for pain relief, as delivered by syringe (d). A similar technique has been used in all pre-clinical animal experiments and clinical studies with human chromaffin cell injections for pain.

Adult human chromaffin tissue has also been transplanted in humans for cancer pain [62], but when the immune response in the human host is examined after human chromaffin grafts, one conclusion is that further purification and/or the immunoisolation of tissues grafted in the CNS will be necessary when using these primary adult human adrenal sources, particularly when the possibility of long-term and repeated grafting is considered [63]. However, there are recent reports [64] of successful human fetal adrenal transplant to treat pain associated with rheumatoid arthritis, and in a rat model of partial nerve injury [65], certainly suggesting that fetal or precursor chromaffin tissue could be used as an antinociceptive source [66, 67]. But using such primary tissue sources for the further purification [68] for successful cell therapy necessitates immunosuppression of the human host, such that examination of encapsulation technologies of grafted cells continued in many rat and human studies (Figure 1).

### 7.2. Clinical Trials Utilizing Cell Therapy for Neuropathic Pain

As mentioned above, cell therapy utilizing intrathecal adrenal chromaffin grafts to treat cancer pain was initiated in the early 1990s [41, 46, 61], which reported long-lasting pain relief, in correlation with met-enkephalin release into the CSF [43]. Typically not all late-stage cancer patients respond well to systemic opioids for pain management, with adverse effects and poor pain control [69], and hence requiring intrathecal delivery. The efficacy of this cell therapy technique depends on the ability of those cells to produce analgesic opioids and on the immuno-privileged property of the central nervous system, in which rejection risks are limited [70]. Before inclusion in an open Phase II trial, all the cancer patients to be grafted with human chromaffin cells had their pain controlled by daily intrathecal (I-Th) morphine administration. Out of the 12 patients who profited from enhanced analgesia with long-term followup (average 4.5 months), five no longer required the I-Th morphine (with prolonged interruption of systemic opioids as well), two durably decreased I-Th morphine intake, and five were stabilized until the end of their followup. Durable decline and stabilization were interpreted as indicative of analgesic activity by comparison with the usual dose escalation observed during disease progression, related to increased CSF met-enkephalin levels associated with the grafts [71]. The grafts were tolerated, and there is evidence of long-term survival [72], despite the presence of CSF lymphocytes, where single treatment failure and three of four cases of partial efficacy occurred in grafts where CSF lymphocytes were present, indicating that impairment of the local immunosuppressive balance can lead to activation of host CSF CD4 T cells and drive a rejection process when grafts are not encapsulated [63]. It was concluded that graft immunoisolation, by using cell encapsulation, seems to be unavoidable in spite of the graft site [70]. Such ultimately failed clinical trials provided a better understanding of the limits (at that time) for this approach [43, 62, 72, 73]. Adult human chromaffin tissue has also been transplanted in humans for cancer pain [62], but when the immune response in the human host is examined after human chromaffin grafts, one conclusion is that further purification and/or the immunoisolation of tissues grafted in the CNS will be necessary when using these primary adult human adrenal sources, particularly when the possibility of long-term and repeated grafting is considered [63]. However there is a recent report [64] of successful human fetal adrenal transplant to treat pain associated with rheumatoid arthritis, certainly suggesting that fetal or precursor chromaffin tissue could be used as an antinociceptive source [66]. But such primary tissue sources for the purification and use of chromaffin cells are not likely to be homogeneous, since they are often obtained from multiple donors. The ability to use and manipulate stable antinociceptive cell lines as a defined source has provided a rich literature for their experimental use in cell therapy (Table 1).

## 8. Strategies for the Creation of Immortalized Cell Lines: Rationale/Studies

### 8.1. Naturally Occurring (Tumor) Cell Lines

A cell line has the ability to be expanded *in vitro*, is stable enough in its phenotype to be characterized *in vitro* and after grafting; and can be used for *in vivo* transplant. The archetypal adrenal medullary cell line is the rat PC12 cell line, first established from a transplantable rat adrenal pheochromocytoma [74], which was shown to respond to NGF with reversible loss of mitotic activity and differentiation to a neuronal phenotype. This natural, oncogenic cell line has been used as a model to bioengineer the addition of the gene [75] for the analgesic [76] peptide histogranin (SHG) which acts as an antagonist for the excitatory NMDA receptor, as SHG can enhance the antinociceptive properties of grafted cells, such as chromaffin transplants [77]. Although originally reported to lack phenylethanolamine N-methyltransferase (PNMT) and epinephrine synthetic capability [74], further characterization [78] suggests both PNMT activity and epinephrine synthesis in PC12 cells. Although this cell line has often been examined for its response to manipulation to agents, such as morphine analogs important in pain modulation [79], it has also been tested as a grafted catecholamine source to test cell therapy for pain relief [80]. However, PC12 cells tend to form tumors, rather than to integrate and release antinociceptive agents. Grafts of the mouse B16 F1C29 melanoma cell line, which also release catecholamines, was able to reduce pain behaviors in the tail-flick model when accompanied by morphine [81], but again, such grafts are tumorigenic, and their transplant can itself induce pain behaviors [82]. The monaminergic human NB69 neuroblastoma cell line was able to reduce neuropathic pain in a nerve injury model [83], presumably related to serotonin release from the grafts, but the tumorigenic potential is a consideration with a non-differentiated tumor line. Other studies with implantation of tumor-derived cell lines, like AtT-20 or AtT20/hENK [84], Neuro2A [85], Neuro2A/POMC [86], or P19 [87], that overexpress opioid peptides have been attempted, but such grafts would also carry the risk of tumor formation.

Although the ability of opioids to provide pain relief with SCI remains controversial, the release of enkephalin-like molecules from genetically modified cell grafts, such as At-T20/ENK cells, as therapeutic was an early strategy [84, 88]. One goal of such a opioid-based strategy would be to reduce the side-effect of tolerance that develops with morphine and its analogs [84] (Table 2).

### 8.2. Conditional Immortalization to Create Cell Lines

Retroviral infection of neural precursors, when cells are actively proliferating, with an immortalizing gene sequence *in vitro*, is a strategy applicable to a variety of cell types that might be useful for transplantation [89], and especially the neural phenotype with v-myc [90] or the wild-type SV40 large T temperature-sensitive antigen (tsTag) oncogene [91]. Immortalization with tsTag can result in cell lines capable of undergoing proliferation at permissive temperature (33°C) and differentiation under appropriate temperature conditions (nonpermissive; 39°C) [92, 93]. Infection of precursors with the temperature-sensitive allele of Tag (tsTag) *in vitro* [94] and *in vivo* [95] has allowed cells to undergo growth arrest and continue differentiation under nonpermissive temperature (39°C) conditions. These differentiating temperatures are possible both *in vitro*, allowing transformed cells to revert to a near-normal primary cell phenotype, as well as *in vivo*, where CNS transplant temperatures are near 39°C [96], and tumors are not formed because the immortalizing gene is not expressed. Thus, conditional immortalization with the oncogenic tsTag construct incorporates the advantages of cell lines, including the convenience of growing large quantities that can be characterized and safety tested and the ability to also genetically engineer-in the expression of additional therapeutic molecules, while reducing the disadvantages of tumor cell lines.

Though describing engineered-cell grafts as “biological minipumps” for secretion of neurotrophic or antinociceptive agents has only been recently discussed [97, 98], the practicality has been examined for at least the last 25 years [99, 100]. But, the same strategy, using engineered cells that might secrete potentially antinociceptive molecules when placed in the lumbar subarachnoid space after PNS or CNS injury, much like the primary adrenal chromaffin cells and opioid cell lines described above, has seen few applications for use in chronic pain [81, 84]. But the potential application of such cell line grafts for the diverse problems with neuropathic pain in human therapy is significant [101], given the paucity of homogeneous primary tissue. Unlike primary or immortalized chromaffin cells, the engineered cells being tested in a variety of models of acute and chronic pain were initially neuronal epithelial precursor cell lines derived from the rat medullary raphe. Two lines that have been bioengineered, called RN46A, and RN33B were isolated from embryonic day 12.5 (E12.5) rat brainstem after immortalization with the SV40 tsTag sequence [102, 103]. Although they were derived from the same primary cultured neuronal precursors, there are significant differences in their phenotypes: RN46A cells are an early serotonergic precursor neuronal cell line, with the potential to switch developmental phenotype [102], depending on the timing and exposure to a variety of neurotrophic and other factors, including BDNF [104], CNTF [105], GDNF [106], and ACTH [107]. This cell line was made to synthesize and secrete the neurotrophin BDNF, by the addition of the sequence for rat BDNF to its genome, causing the cells to have improved survival *in vitro* and *in vivo*, and develop a permanent serotonergic (5HT) phenotype [108]. Since additional 5HT might be postulated to have a beneficial antinociceptive effect on neuropathic pain [109] differentiated cells were placed in a lumbar subarachnoid location after sciatic nerve chronic constriction injury (CCI). Transplants of this serotonergic cell line 46A-B14, placed two weeks after CCI and the development of severe hypersensitivity to thermal and tactile stimuli were able to potently and permanently reverse the symptoms of neuropathic pain [110], compared to grafts of the same cells which did not receive the BDNF gene and did not synthesize 5HT *in vitro* or *in vivo*. Transplants of other cell lines genetically engineered to synthesize and secrete potentially antinociceptive molecules such the inhibitory peptide galanin [111], the neurotrophin BDNF [112], and the inhibitory neurotransmitter GABA [113] have all been tested after CCI and the induction of neuropathic pain, and each has reversed the thermal and tactile allodynia and hyperalgesia that develop after CCI. Each engineered cell line is characterized for its particular gene expression under permissive and nonpermissive temperature conditions, since the cell lines are usually transplanted immediately after proliferation at 33°C. Following placement of the differentiating cells in the subarachnoid space, especially in models of pain, both cell graft survival and continued expression of the antinociceptive phenotype were examined *in vivo*. An example of such an engineered rat neuronal cell line, the RN33-GAD67, which synthesizes and secretes GABA after differentiation *in vitro* and transplant *in vivo* [113] in the CCI pain model, where GABA is synthesized after differentiation in these cells. But such an effect for neuropathic pain seems to require an early transplant time, since grafts of these rat neuronal GABA cells are less effective when placed late after nerve injury [114]. With both types of behavioral hypersensitivity, thermal and tactile hyperalgesia, rat GAD67 grafts cause immediate reversal of hypersensitivity when the behaviors are measured one week later. Such potent reversal is common to each of the engineered cell lines used for therapy after partial nerve injury models, and more recently with SCI models [115–118], especially thermal hyperalgesia. But using the CCI model of neuropathic pain and near-identical transplant numbers and experimental conditions for all these studies has identified the rat GABA- and 5HT-cell lines as especially efficacious, since attenuation of hypersensitivity is more potent and permanent in the presence of these graft phenotypes, although early transplant time seems to be favored in the CCI model [119]. But other antinociceptive cell types have also been conditionally-immortalized to test their usefulness in eventual clinical applications.

Mitotic cells found in embryonic medullary adrenal tissue can also be conditionally immortalized with the tsTag oncogene so that the differentiated cell type keeps many of the phenotypic features of primary chromaffin cells. Conferring immortalization with the SV40 large T antigen expression has a variety of effects on cells when the wild-type large T protein is present, including binding of large T and inactivation of the growth suppressors pRB, p53, and SEN6 [120, 121], a decrease in G1 and increase in G2 and M cell cycle phase duration [122], and the ability of large T antigen to block the differentiation process [123]. However, after immortalization with the temperature-sensitive allele tsTag [94, 124], immortalized cells resume the stage of life span and function of an uninfected cell when they are shifted to nonpermissive temperature conditions [125]. These cells at the nonpermissive temperature have lost the ability to drive cell proliferation, since the large T antigen is labile at the higher temperature conditions [126] and the T antigen is not able to drive mitosis in cells immortalized with the construct, and differentiation is favored [94, 124]. In general, SV40 large T antigen-immortalized cell lines retain the phenotype of the differentiated lineage of the parent. Cell lines generated with the SV40 large T antigen retain contact inhibition *in vitro* [127, 128] and do not produce tumors or induce immune rejection even when injected into nude mice [129] or rats [130–137].

Rat and bovine chromaffin cells immortalized with tsTag *in vitro* [138] express many of the markers found in primary chromaffin cells and when differentiated *in vitro*, as the oncogenic Tag protein is degraded and mitosis ceases, these markers remain and are able to be regulated by continued differentiation, by agents such as dexamethasone and by stimulation of the cAMP pathway with forskolin, mechanisms seen in primary chromaffin cells [138]. Such immortalized chromaffin cells are stable and appear homogeneous, suggesting that they could be useful for further genetic manipulation and as a source for transplant studies *in vivo* [139].

The cell biology and developmental responsiveness during differentiation of chromaffin cells [140] reveals clues to the differentiation program of conditionally immortalized chromaffin cell lines *in vitro*. The enzyme tyrosine hydroxylase (TH; EC1.14.3.x) catalyzes the rate-limiting step [141] in the biosynthesis of catecholamines in chromaffin cells in the adrenal medulla [142, 143] and has been used as one of the antigenic markers for the mature chromaffin phenotype of primary rat and bovine chromaffin cells *in vitro* [144], as well as D*β*H and PNMT. Both the rat RAD5.2 and bovine BADA.20 chromaffin cell lines express these catecholamine enzyme immunoreactivities at both permissive (low levels) and nonpermissive temperatures, when the cells are proliferating or differentiating, respectively, though levels of the D*β*H enzyme appears to change with differentiation at nonpermissive temperature (39°C). Tyrosine hydroxylase (TH) expression is not upregulated in the rat chromaffin cell line but seems to be a feature of immortalized bovine chromaffin cell *in vitro* [138]. But further increased catecholamine enzyme expression in the chromaffin cell lines requires treatment with forskolin and/or dexamethasone during differentiation, since differentiation at 39°C is in serum-free medium [138]. Differentiated primary chromaffin cells from rat [145, 146] and bovine [34] sources have often been used to study the synthesis and release of the catecholamine neurotransmitters norepinephrine and epinephrine *in vitro*. However, even with upregulation of enzyme expression, these conditionally immortalized chromaffin rat and bovine cells do not synthesize catecholamines under *in vitro* conditions [138]. Since chromaffin cell lines probably require an adequate substrate interaction for a completely normalized chromaffin phenotype, the absence of detectible catecholamine synthesis in differentiated RAD5.2 and BADA.20 cells may be due to removal from their fibroblast environment. Another possible, and more likely, explanation for the absence of catecholamine synthesis is a continued low level of Tag expression, even though it is greatly reduced after three weeks of differentiation at 39°C. It is possible that even a low level of Tag suppresses some normal cellular functions, such as neurotransmitter synthesis.

This attempt at conditional immortalization of chromaffin cells using the tsTag oncogene and retroviral infection *in vitro*, demonstrating continual cell lines retaining many features of the mature chromaffin cell phenotype. The availability of conditionally immortalized chromaffin cell lines for a variety of studies, including their use as transplants in various models of neuropathic [139], reflects the growing interest in the development of molecular biological techniques of cellular therapy for treating neuropathic pain, but further attempts to develop the immortalization technologies were needed.

### 8.3. Reversible Immortalization to Create Cell Lines

The ability to reverse immortalization in a tightly controlled manner was the logical next step in the creation of cell lines from rare phenotypes [147]. But such reversible-immortalized cell lines that might be used for antinociception have been little studied [148]. The generation of chromaffin cell lines, utilizing the temperaturesensitive allele of SV40 large T antigen (tsTag) are able to reverse neuropathic pain after transplant in the spinal subarachnoid space after CCI of the sciatic nerve [139]. Even with near 100% disappearance of Tag in the grafts within a few weeks after transplant [139], oncogene expression *in vivo* remains a potential possibility and such cells would not be an appropriate strategy for safe clinical use in humans.

Studies exploiting sitespecific DNA recombination and Cre/lox excision have suggested that cells can be targeted *in vitro* [149] and *in vivo* [150] for removal of deleterious genes, including the Tag sequence [151]. Reversible immortalization with Tag and Cre/lox technology was first reported with human fibroblasts by Westerman and Leboulch [152] and more recently with human myogenic cells and hepatocytes [153] and hepatic progenitors [154]. In these latter studies, Cre was introduced by transfection or infection, inefficient methods that may lead to a lack of disimmortalization and the loss, through the subsequent selection of disimmortalized cells, of a significant part of the population. Moreover, *in vivo* excision is not possible. Use of a vector that allows a silent, but inducible, form of Cre is preferred for the timed excision of the oncogene.

A number of chimeric Cre-containing fusion proteins, especially fusions with the ligand-binding domains of steroid receptors, have been created to utilize the binding by synthetic ligands to activate Cre [155]. CrePR1 is a fusion protein [156], consisting of the fusion of Cre and the ligand binding domain of a mutant human progesterone receptor (hPRB891). Cre activity in the cells is activated by the binding of the steroid RU486, which in turn induces the translocation of CrePR1 to the nucleus where the Cre is active to excise the floxed sequences. The requirement for RU486 and the use of a mutated steroid receptor for disimmortalization would assure that if nondisimmortalized cells were transplanted, Cre would not be activated by circulating endogenous progesterone, a strategy used for inducible recombination with *in vivo* CNS studies [157].

It has been demonstrated [158] that embryonic rat adrenal chromaffin cells could be immortalized with a oncogenic tsTag construct, utilizing retroviral infection of these early chromaffin precursors, where the tsTag construct (tsA-TN) was flanked by loxP sequences. Following isolation of immortalized cells using positive neomycin selection, the cells were further infected with a retrovirus expressing the CrePR1 gene, which encodes a fusion protein which combines Cre activity plus the mutant human steroid receptor, hPRB891. Cultures of embryonic rat adrenal cells were immortalized with the tsA-TN retroviral vector encoding the loxP-flanked temperature-sensitive allele of SV40 large T antigen (tsA-TN), which included a positive/negative neo/HSV-TK sequence for selection with either G418 or gancyclovir, respectively.

A number of chimeric Cre-containing fusion proteins, especially fusions with the ligand-binding domains of steroid receptors, have been created to utilize the binding by synthetic ligands to activate Cre [155]. CrePR1 is a fusion protein [156], consisting of the fusion of Cre and the ligand binding domain of a mutant human progesterone receptor (hPRB891).

When immortalized chromaffin cells are disimmortalized with cre-lox technology to disimmortalize the chromaffin cells *in vitro*, complete removal of the Tag sequence before differentiation seems to allow neurotransmitter synthesis and a more normal phenotype [158]. Compared to downregulation of the tsTag protein in conditionally immortalized rat chromaffin cells, disimmortalization *in vitro* in these disimmortalizable rat chromaffin cells, called the loxtsTag/CrePR1/RAD chromaffin cell line, the Tag protein was completely and efficiently removed by 10 days of treatment with RU486, followed by incubation with the antibiotic gancyclovir [158]. Cells which were not disimmortalized, were removed by their continued expression of the thymidine kinase (TK), which is toxic in the presence of gancyclovir [159].

Irreversible removal of a potentially subverting oncogene by its excision using the Cre/Lox system might thus be a clinically useful strategy, especially since the core temperature of humans is lower than that of rodents, and the expression of a temperature-sensitive antigen might not be completely blocked in a clinical context [152, 160–164]. Note that in this respect, use of moduletable Cre activity that can be activated by the synthetic steroid RU486 [156, 157] has added a means to select the timing of disimmortalization and render the overall procedure more flexible and efficient. Interestingly, the disimmortalized rat chromaffin cells had very increased expression of tyrosine hydroxylase (TH), the rate limiting enzyme for catecholamine synthesis, *in vitro*. This was accompanied by 5-fold increase in norepinephrine synthesis *in vitro* [158]. But these disimmortalizable rat chromaffin cells not only synthesize epinephrine after Tag excision, but they also apparently make increased catecholamine enzymes besides TH, judged by qualitative immunohistochemistry for the enzymes compared to both nonexcised and those immortalized with only tsTag [138]. Also of importance, transplant of disimmortalized rat chromaffin cells was able nearly eliminate neuropathic pain in the CCI model of partial nerve injury, when compared to the injury alone or transplant of immortalized chromaffin cells. Rather than suggesting that antinociception is the result of catecholamine synthesis, release or secretion from grafted chromaffin cells, the existence of an equivalent functional effect by nondisimmortalized cells suggests that another agent or mechanism is responsible for reduction of neuropathic pain by these genetically manipulated chromaffin cells, at least in this model of pain. Even if chromaffin grafts do not make significant levels of catecholamines *in vivo*, the antinociception the grafts provide might be a result of other antinociceptive molecules synthesized and released by the cells, such as GABA or met-enkephalin. Presumably the increased norepinephrine phenotype recovered following excision of the oncogene by disimmortalized cells would function to advantage in cell therapy, but with disimmortalized rat chromaffin cell grafts no such advantageous effect could be demonstrated. Rather, the value of disimmortalization before transplantation is to provide a measure of safety, with the complete absence of the oncogene and prevention of even a remote possibility of viral transfer of the large T antigen in the host, after grafting such cells (Table 3).

### 8.4. Transgenic Opioid Expression in Immortalized Cell Lines

A further advance to model genetically modified, disimmortalizable chromaffin cell lines, is the work by Duplan and colleagues [165], who infected the disimmortalizable loxtsTag/CrePR1/RAD chromaffin cell line with constructs for the synthesis and secretion of the opioid met-enkephalin (met-Enk). These transgenic rat chromaffin cell lines expressed easily detectible met-ENK *in vitro* cells, which contained the met-ENK construct contained high levels of this opioid. The transgene also contained a neurotrophin growth factor (NGF) sequence for secretion of synthesized nascent protein, and chromaffin cells which contained the met-ENK transgene were able to secrete the highest levels of the met-ENK opioid from the cells. The value of opioids from chromaffin grafts in cellular therapy, especially for pain [166], has seen precedents in both animal [32, 62, 167], and more recently, human clinical work [43, 71, 165] when primary chromaffin tissue was used as a graft source. When these disimmortalizable loxtsTag/CrePR1/RAD chromaffin cells were grafted, by Duplan and colleagues, two weeks before injection of formalin into the hindpaw in a model of tonic pain [168, 169], those rats which had been given grafts of cells which secreted met-ENK did not develop the long-term response to formalin injection, compared to rats which had no grafted cells or those that had only received cells which were transgenic for the vector only [170]. Although it is not yet known how disimmortalization may influence the expression of transgene, such as the opioid met-ENK gene used here, irreversible removal of a potentially subverting oncogene by its excision using the cre/lox system might be a clinically useful strategy. Of course, immortalization of human chromaffin tissue with an oncogene, such as SV40Tag, is not likely with any potential for deleterious expression of SV40 proteins [171], but disimmortalization utilizing cre/lox site-directed removal of oncogenes in a growing technology to create useful graft sources for cell therapy for a variety of conditions [160, 161, 172]. There are a variety of possible oncogenic sequences that could be used for the reversible immortalization of human chromaffin cell lines, including v-myc [173]. However, the creation of reversibly immortalizable human chromaffin cell lines, perhaps from precursors [174], is still somewhat in the near future [175]. But such a homogeneous source will also allow for the manipulation of the chromaffin cell's genome to investigate the mechanisms of action responsible for cell grafts to repair the injured CNS environment. Similar immortalization of human chromaffin precursors and creation of human chromaffin lines [65, 67, 73] presage the advent of cellular therapy as a therapeutic strategy that includes further development of human stem cell and progenitor/precursor cell lines [176].

## 9. Current Strategies for Immortalized Cell Lines: Rationale/Studies

### 9.1. Stem Cells

An increasing number of articles describing regenerative methods to improve function after injury and in certain disease states have appeared in the last few years. Most are related to transplants with stem cells [98], progenitors [176, 177], and bone marrow and nontransplants. Stem cell transplants can be ranked in the following descending order of preference; bone marrow-derived cells, neural stem cells, human umbilical cord blood cells, embryonic stem cells, and myoblasts. Bone-marrow-derived cells and human umbilical cord blood cell have been used for study in various disease fields. The nonstem cell transplantation group is made up primarily of islet cells, followed by biomaterials, and other cells or tissues from a variety of sources [178]. With their more limited multipotency, the use and potential of progenitor cells for improving function has still made significant progress recently [179–181], especially in the potential for renal and cardiac regeneration and reduction of ischemia [181–183]. But another critical potential to be fulfilled is in the area of the management of chronic, and especially neuropathic, pain.

### 9.2. Stem/Progenitor/Precursors (Animal Studies)

In a recent report [184] utilizing the partial nerve injury with CCI to induce neuropathic pain, rat spinal embryonic progenitor cells (SPC) that used basic fibroblast growth factorB2 (FGF-2) for proliferation of the SPC *in vitro* were able to reduce thermal hyperalgesia after intrathecal transplant. Presumably, grafted cells had been induced to a GABAergic phenotype by FGF-2 *in vitro* and survived in its absence after transplant, maintaining their phenotype to modulate the neuropathic pain. The authors suggest that the grafts also increased the glycine content in the CSF of grafted animals, suggesting that if precursors could be induced to a phenotype that provides nociceptive inhibition, they would function much like cell minipumps, surviving in the intrathecal space. Also in the CCI model of peripheral pain, freshly isolated syngeneic marrow mononuclear cells were injected i.v. following the unilateral nerve injury and tactile allodynia and thermal hyperalgesia evaluated weekly. Marrow transplantation did not prevent pain, and 5 days after CCI all animals were equivalently lesioned. However, 10 days after CCI, rats that received marrow transplants demonstrated paw withdrawal response and paw withdrawal latency patterns indicating recovery from pain, whereas untreated rats continued to have significant pain behavior patterns. The mechanisms underlying this improvement following bone marrow injection are unknown. The authors speculate that the marrow cells functioned as anti-inflammatory, neuroprotective, and proangiogenic, modulating ischemic, inflammatory, and cytotoxic events in the pain that follows nerve constriction in this model. However, marrow transplants are also known to exacerbate diabetic neuropathy in a different model of pain [185]. In this case, marrow cells fused with peripheral neurons, stimulating apoptosis.

 One cause of severe neuropathic pain is traumatic injury that involves SCI is spinal root avulsion, and replacement of DRG neurons could reduce that pain. A recent study investigated whether human neural stem/progenitor cells (hNSPCs) transplanted to the DRG cavity can serve as a source for repairing lost peripheral sensory connections [186]. The hNSPCs robustly differentiate to neurons, which survive long-term transplantation. The neuronal population in the transplants was tightly surrounded by astrocytes, suggesting their active role in neuron survival. Furthermore, 3 months after grafting, hNSPCs were found in the dorsal root transitional zone (DRTZ) and within the spinal cord. The level of differentiation of transplanted cells was high in the core of the transplants whereas cells that migrated to the DRTZ and spinal cord were undifferentiated, nestin-expressing precursors. However, hNSPCs are not sufficient to restore normal sensory function; additional factors are required to guide their differentiation to the desired type of neurons.

### 9.3. Neuroprogenitor Cell Lines for Pain (The NT2 Cell Line)

More than two decades ago, it was discovered that, when treated with retinoic acid (RA), a human embryonal carcinoma cell line, NTera2cl.D/l (NT2, hNT2), differentiates irreversibly into several morphologically and phenotypically distinct cell types, which include terminally differentiated postmitotic CNS neurons [187, 188]. Successive replating of RA-treated NT2 cells, in the presence of growth inhibitors, results in the isolation of purified human neurons [189], which have been extensively characterized and tested *in vivo* in a number of animal models of traumatic injury and neurodegenerative disease [188, 190–194]. This NT2 human neural cell line has been used for a variety of studies that reveal not only the regulation of an oncogenic phenotype by agents such as retinoic acid [189, 195, 196], but it has been well characterized for the expression of a variety of neural phenotypic properties [197] and proteins [198, 199] with differentiation of the cells *in vitro* and *in vivo* [200]. The potential application of NT2 neurons in cell transplantation therapy for CNS disorders, and their use as vehicles for delivering exogenous proteins into the human brain for gene therapy, has been envisioned [201]. Such NT2 neurons have been used in Phase I-II clinical trials for the treatment of stroke [202–204], and this cell line or its derivatives can likely be utilized for further reparative transplant strategies [205]. The rate-limiting enzyme GAD, for GABA synthesis is present in differentiating NT2 neurons *in vitro* [206, 207], and GABA is a phenotype for NT2 cells differentiated and transplanted *in vivo* [208]. But the NT2 cell line has a great variety of phenotypes expressed in differentiated cells [193, 207], making it less-than-ideal for a specific antinociceptive phenotype expression that might be required for application in pain management, as has been modeled in rat cell lines described above. While induction of a GABAergic phenotype in neural stem cells is possible with a somewhat complicated method of sequential exposure to epigenetic signals *in vitro* [209], the host graft environment does not always allow for induction of desirable phenotypes *in vivo* [210]. A naturally occurring, stable antinociceptive phenotype in a clinically useful human progenitor cell line, such as that derived from the NT2 cell line, is more desirable, and these are described below.

### 9.4. NT2-Derived Cell Lines for Pain

Since the NT2 cell line contains a mixed phenotype population of cells, many of which would likely be antinociceptive based on multiple studies with rat cell lines by this author and others using central and peripheral models of neuropathic pain [110, 111, 113, 115–119, 170, 211], it was considered likely that individual cell lines could be subcloned from the NT2 parental cell lines, using ordinary subcloning techniques involving isolation of individual cells plated sparsely, allowing them to grow into colonies, surrounding these with cloning rings, and removing these colonies to establish individual cell lines. This rather laborious process resulted in a number of well-growing, morphologically and immunohistochemically distinct NT2 subclonal cell lines, numbered consecutively as they were isolated. Two of the cell lines were chosen for their potential to function as sources of neurotransmitters which might prove useful in further testing in animal models of pain, the hNT2.17 [212] and hNT2.19 [213] cell lines. Since they are derived from the neuroprogenitor parent cell line NT2, these are considered to be human neuroprogenitor cell lines as well, resulting in a neuronal-limited phenotype, and will be described below. These human progenitor cell lines are being developed for clinical use. Their characterization and use in animal models reflect what will be required of any similar regenerative cell therapy for FDA approval [98, 214].

### 9.5. The Human Neuronal GABA hNT2.17 Cell Line

Centrally induced excitotoxic SCI has been developed as a model of neuropathic pain [215, 216]. Intraspinal injection of quisqualic acid (QUIS), a mixed AMPA/metabotropic receptor agonist, produces injury with pathological characteristics similar to those associated with ischemic and traumatic SCI [217]. In addition, the pathological changes that this SCI induces, significant mechanical allodynia, and thermal hyperalgesia have been shown to be important behavioral components, without the additional motor dysfunction seen in other SCI models [218]. Each of these sensory behaviors is indicative of altered sensory function and/or pain, similar to that reported after SCI. After spinal transplantation of primary adrenal tissue grafts following QUIS injections, pain-related behaviors, including the hypersensitivity to mechanical stimuli and “excessive grooming” were significantly reduced [219]. Given the related loss of GABA inhibition that seems to accompany SCI and the induction of neuropathic pain [220–222], the excitotoxic SCI model was used to examine the hNT2-derived GABAergic hNT2.17 cell transplant into the lumbar subarachnoid space following injury and the ability of those grafts to reverse behavioral hypersensitivity [212]. These cells cease to express tumor genes, express an exclusively neuronal, GABAergic and glycinergic phenotype, and synthesize, secrete and release GABA and glycine into the extracellular environment with differentiation [212]. Their morphology is similar to the GABA/glycine spinal interneurons found in the dorsal horn sensory laminae [223] and such characteristics are stable in more than 10 years of use in transplant studies. These inhibitory human neurons additionally co-localize GABA and glycine and the vesicular inhibitory amino acid transporter (VIATT, VGAT), especially along the neurite outgrowths *in vitro*, suggesting that this molecular machinery allows co-release in hNT2.17 cells [224], without the need for a separate glycine transporter. When differentiated hNT2.17 cells are placed two weeks after the QUIS SCI, mechanical allodynia and thermal hyperalgesia are potently and permanently attenuated, with no greater effect when twice the normal transplant dose (1 million cells/i.t. injection) is used [225, 226], or grafts are placed in the cervical subdural space [226]. Besides transplant dose and graft placement, the immunosuppression regimen and transplant time after SCI were also optimized [226]. The same optimal transplant dose was only moderately effective when placed in chronic SCI, six weeks after SCI, compared to 100% effectiveness when placed in an acute SCI, 2 weeks after injury. Additionally, maximal graft effectiveness required two weeks of immunosuppression with cyclosporine A (CsA; 10 mg/Kg), immediately following transplant. No immunosuppression or less lengthy exposure to CsA provided minimal or no attenuation [226]. The “excessive grooming” behaviors associated with this model were also examined. When excessively grooming rats that had been transplanted with either viable or nonviable hNT2.17 cells and exposed to different immunosuppression regimens were examined for development, resolution, worsening, or no change of excessive grooming, a trend toward improvement was associated with viable grafts and at least 1 week of accompanying CsA immunosuppression. When transplant was delayed to 6 weeks, no improvement in excessive grooming was seen. This last finding duplicates what was seen in the QUIS SCI model and graft of antinociceptive adrenal medullary tissue [219], suggesting potent reversal of behavioral hypersensitivity may have a neuroprotective effect on the progression of spinal excitotoxicity associated spinal lesions. We [227], and others [228], have recently reported on the use of hNT2.17 cell therapy in various other models of peripheral and central nervous system damage, including: CCI of the sciatic nerve, streptozotocin-induced diabetic peripheral neuropathy (DPN) pain, and severe contusive SCI. Much as we [229] and others [56] have seen in the CCI peripheral nerve injury model and antinociceptive cell grafts Vaysse and colleagues [228], reported that the decrease in GABA expression in the spinal dorsal horn of CCI injured animals is concomitant with a decline of its synthetic enzyme GAD67 immunoreactivity (ir) and mRNA but not GAD65. In hNT2.17 transplanted animals a strong induction of GAD67 mRNA one week after graft was seen, which was followed by a recovery of GAD67 and GABA ir. This effect paralleled a reduction of hindpaw hypersensitivity and thermal hyperalgesia induced by CCI. These results suggest not only that hNT2.17 GABA cells can modulate neuropathic pain after CCI by minimizing the imbalance and restoring the cellular GABAergic pathway, but that such a mechanism may be associated with any potent antinociceptive cell graft, at least in the CCI model. The same, or a similar mechanism may explain the antinociceptive effects of hNT2.17 grafts in contusive SCI and DPN pain [227]. DPN pain studies have suggested aberrant spinal or supraspinal modulation of sensory processing [230, 231], including a central mechanism [232] with the ventral posterolateral thalamus becoming hyperexcitable in the presence of spinal and supraspinal disinhibition. Disinhibition and loss of spinal GABA modulation are also well reported in SCI pain [222]. But evidence for a GABAergic mechanism associated with hNT2.17 transplant and antinociception in these other models of pain awaits further studies.

### 9.6. The Human Neuronal 5HT hNT2.19 Cell Line

Current understanding of central and supraspinal [233] mechanisms for the induction and maintenance of chronic pain after SCI suggests a major role for the hypofunction of serotonergic (5HT) inhibitory systems [234–236]. This same SCI leads to the loss of descending serotonergic excitatory inputs caudal to the lesion site and altered neurotransmitter status within the ventral horn a-motoneurons, which also contributes to motor dysfunction after SCI [116, 237]. A variety of animal studies have used a 5HT rat cell line [110, 116–118] or 5HT raphe transplants [238, 239] as a means to ameliorate some of these problems. Supplemental cell therapy can also work to create a spinal environment to ameliorate local damage and simultaneously promote a regenerative response in multiple axonal populations, including descending spinal serotonin fibers [240], or reverse chronic pain after SCI by reversing the hyperexcitability in the dorsal horn pain processing centers [117]. We have described the use of 5HT cell therapy with a rat 5HT cell line that is able to permanently reverse neuropathic pain that develops after partial nerve injury [110] and hemisection SCI [115, 241]. The human neuronal 5HT hNT2.19 cells used for cell therapy after severe contusive SCI reverses behavioral hypersensitivity [241], without affecting motor dysfunction when grafts are placed intrathecally. These same cell grafts modestly recover motor function when placed intraspinally [213] in the same severe contusion SCI model of chronic pain and motor dysfunction. Additionally, grafts of hNT2.19 cells attenuate tactile allodynia and thermal hyperalgesia in the excitotoxic SCI QUIS model [227], much like grafts of hNT2.17 cells. In fact, lumbar intrathecal 5HT hNT2.19 and GABA hNT2.19 grafts are equally nociceptive no matter which SCI pain model is used, excitotoxic or contusive [227], suggesting that these cells may affect the same or similar mechanism-of-action that is common to both models that initiates behavioral hypersensitivity. We have already shown a GABAergic mechanism-of-action for grafts of hNT2.17 cells [228] and suggested it may be common in SCI pain. Although grafts of a 5HT rat neuronal cell line which is antinociceptive after hemisection SCI [116], depending on graft location [115], much like grafts of hNT2.19 cells, it does so by attenuating bilateral hyperexcitability of dorsal horn neurons [117], restores spinal serotonin, downregulates the serotonin transporter, and increases BDNF tissue content in the spinal cord [118], these same 5HT rat cell line grafts also induce a GABAergic mechanism of action in the CCI model of nerve injury and neuropathic pain [229]. Obviously, it will be important in future studies to understand how each separate human neuronal cell line provides antinociception in each PNS or CNS pain model, but the same or similar mechanisms are not out of the question. Since the 5HT hNT2.19 cells, like the hNT2.17 cell line, are exclusively neuronal, although with a different neurotransmitter phenotype, and equally nontumorogenic before and after transplant [227, 241], this human progenitor cell line is equally appropriate to develop as a clinical tool, not only to treat neuropathic pain, but also motor dysfunction, especially after SCI [241–243] (Table 4).

## 10. Summary of Advantages and Disadvantages and Future Directions of Cell-Therapy Approaches

To summarize the conclusions from 30 years of cell therapy studies, the advantages and disadvantages of a cell-based approach to the treatment of neuropathic pain would include the following (1) It is likely that only human cells will be useful as a source, whether primary tissue or cell lines given that such sources are the least likely to be rejected, would function appropriately, and respond to environmental cues in the human host. Encapsulation technologies could be helpful here, if these technologies could keep the grafts both viable and functional. However, (2) it is likely that there are limits to the achievable levels of a given biologic agent that can be delivered by the cells and multiple intrathecal injections over time, with return of pain, may be necessary. (3) It is possible that delivery of a multitude of substances, in addition to those of therapeutic interest, many of which cannot be completely defined before hand, will be associated with cell-based therapy. Different subgroups of pain patients may respond to such agents in either positive or negative ways dependent on each person's primary cause of pain. Valid and reliable phenotypic classification of pain based on individual signs and symptoms, and various biomarkers, may be helpful in defining such subgroups and their responses to a specific substance related to cell transplant. Such responses are unknown until very large populations are treated with any given cell source. (4) It seems that more acute neuropathic pain is treatable with cell therapy; chronic pain may require multiple i.t. injections as needed. Even still, pain relief may not be equally effective in all cases, and additional pharmacologic and cognitive/adjuvant therapies will be needed. (5) There is a dependence on the survival of implanted cells, which may be limited by immunologic factors, nutrient and oxygen supply, and so forth. However, such survival and efficacy can be tested preclinically, in nonhuman studies. (6) It is likely, at least for now, that only the simplest approaches to creating cell sources will be quickly approved for clinical trials, that is, not overly-manipulated (in cell culture) or bio-engineered cells (containing viral vectors). (7) Some course of immunosuppression will likely be required, even for autologous sources, but such regimens could be tested rigorously in preclinical experiments, that is, nonhuman primates. (8) An intrathecal graft site would likely be the best for cell injections for the treatment of neuropathic pain. Any other transplant type would need to be placed as near to its “site-of-action” as is reasonable, especially if grafted cells are known to not migrate, such as with NT2 cells. (9) If cells are used for antinociception, and placed intrathecally, those that passively secrete inhibitory (or drive inhibitory systems) neurotransmitters would likely work the best, rather than cells that secrete any number of known and unknown agents. (10) Transplant sources need to be tested in as many preclinical peripheral and central models of motor and sensory injury as possible, to avoid later “off-label” use/side-effects in humans. (11) A pragmatic, rather than a purely mechanistic, approach can be used for preclinical work. It is more useful that cell therapy approaches are tested, without necessarily understanding how they work, as long as such technologies are proven as safe as possible. (12) All efforts should be taken to keep patients/provider costs as low as possible, so that cell therapy can be applied almost as readily as pharmacologic treatments. Cell-based research and development will likely be an expensive and complicated solution to treat pain, compared to a purely pharmacologic or mixed-use mechanical-pump/spinal-stimulator delivery approach. (13) The rapid establishment of a Research Ethics Consortium should be established, to be tasked to assemble an interdisciplinary panel of experts who will apply ethical principles to analyze the social merit relative to the economic incentives of this emerging technology [244]. This consortium will evaluate how these novel ethical issues in emerging technologies are addressed under current oversight and regulatory structures and where there may be gaps and need for revised or new public policy approaches.

## Figures and Tables

**Table 1:** Primary tissue used for cell therapy.

Source	Pain model	Results
*Primary cells/tissue: * Adrenal-rat [49, 57, 58, 219, 245–249] Bovine [35, 59, 247, 250–254] Encapsulated bovine [247, 255–258] bovine scaffolds [259] Porcine [253, 254, 260–262] Encapsulated porcine [262] Human [62, 67, 71, 263, 264] Human encapsulated [67]	Acute [247] Midbrain [38] Formalin [54, 58, 247, 249, 253, 260, 261] Nerve injury [46, 57, 67, 256, 262] Dorsal rhizotomy [246] Excitotoxic SCI [219, 255, 265] Hemisection SCI [245] Human Cancer [64, 71, 266, 267] Arthritis [56, 65, 268]	(i) Reduced “excessive grooming” behaviors [219] (ii) Reduction or stabilization in complementary opioid intake in human cancer [71] (iii) Reductions in both fore- and hindlimb mechanical and thermal allodynia [245] (iv) Failed antinociception after intraventricular transplant [256] (v) Reduces edema, anterograde axoplasmic transport [249] (vi) Restores spinal GABA-ir decreased spinal c-Fos [58, 253] (vii) Failed antinociception [247, 247, 269–271] (viii) Reduced cold or TA/TH behaviors [67, 219, 262] (ix) Reduced tonic pain behaviors [261] (x) Delayed, reduced self-directed pain behaviors [246] (xi) Antinociceptive effects on A-delta and C-fiber-mediated responses [59] (xiii) Long-term proenk and tyrosine hydroxlylase in grafts [248] (xiv) Reduce forelimb/hindlimb allodynia [245]

**Table 2:** Naturally occurring (tumor) cell lines.

Source	Pain model	Results
*Tumor Cell Lines: * Rat PC12 [74] Encapsulated PC12 [272] Mouse B16 [81] Human NB69 [83] AtT-20 [84, 273] Encapsulated AtT-20 [85], Neuro2A [85] Encapsulated Neuro2A [1327] P19 [87],	Tail-flick or chemical induction [81, 84, 85, 87, 88] Acute [81, 84, 85, 88, 273] Partial nerve injury (CCI) [83, 272, 274] Formalin [87]	(i) Analgesic [85, 88] (ii) Reduced opioid tolerance [84] (iii) Antinociceptive [81, 83, 84, 273, 274] (iv) Reduced cold allodynia [83, 272]

Bio-engineered—AtT-20/hENK [84, 273] Encapsulated Neuro2A/POMC [86] Autologous rat macrophages/proENK [274] PC12/SHG peptide [75]	Tail-flick or chemical induction [84, 273] Formalin [75]	(i) Increased ACTH release with TET-ON stimulation [86] (ii) Reduced Phase II formalin-induced responses [75]

**Table 3:** Strategies for creating cell lines.

Source	Model	Results	Antinocicptive Molecule Released
*Conditionally immortalized Cell lines: * Embryonic rat Raphe/SV40tsTag [102, 103, 275–277] Embryonic rat DRG neuron, 50B11 [90] Human DRG neuron, HD10.6, v-myc [278] Embryonic rat and bovine Chromaffin [138, 139] Human embryonic Chromaffin cells/tsTag [279]	partial nerve injury (CCI) [139]	(i) Expressed capsaicin receptor transient receptor potential vanilloid family-1 (TRPV-1) and responded to capsaicin *in vitro* [90] (ii) Expressed sensory neuron-associated transcription factors and exhibited capsaicin sensitivity [278] (iii) Antinociceptive [139]	(i) enkephalin? [279, 280]

Bioengineered rat Raphe/tsTag/BDNF [110, 211] Rat raphe/tsTag/galanin [111] Rat raphe/tsTag/GAD67 [113] Rat chromaffin/tsTag/Met-ENK [165, 170]	Partial nerve injury (CCI) [84, 110, 113, 158, 211, 229] Formalin/c-fos induction [170] Hemisection SCI [115–118]	(i) Antinociceptive [84, 110, 113, 116, 119, 158, 211] (ii) Restores dorsal horn GAD/GABA system in CCI [229] (iii) Only intrathecal, not intraspinal, grafts of 5HT cells are antinociceptive [115] (iv) Attenuates bilateral DH hypersensitivity [117] (v) Restores spinal serotonin, downregulates the serotonin transporter, and increases BDNF tissue content [118] (vi) Reduce formalin-evoked c-fos expression [170]	(i) 5HT [110, 116–118, 211, 229] (ii) BDNF [110, 118, 211] (iii) GABA [110, 113] (iv) galanin [111] (v) Met-enkephalin [170]

*Reversibly immortalized Cell Lines: * Tetracycline-regulated of SV40 large T-antigen (Tag) in human embryonic stem (ES) cells and mice [147] Cre/lox-regulated Disimmortalizable Embryonic rat chromaffin cells [158]	Partial nerve injury (CCI) [158]	(i) Antinociceptive [158]	(i) enkephalin? [158] (ii) release norepinephrine [158]

**Table 4:** Stem/Precursor cell lines.

Source	Model	Results
*Stem/Progenitors: * Rat spinal (embryonic) progenitor cells [184] *Adrenal progenitors*—human [281]	Partial nerve injury (CCI) [184]	(i) Reduced thermal hyperalgesia [184]

*Human neuronal/progenitors: * Human NT2 cell line [205, 282, 283]	Excitotoxic SCI pain [283]	(i) Release cannabinoids [282] (ii) Antinociceptive [283]

Human NT2.17 GABA cell line [212, 225, 226, 228, 283, 284]	Excitotoxic SCI (QUIS) [212, 225, 226, 283–285] Streptozotocin-induced diabetic peripheral neuropathy (DPN) [283] Partial nerve injury (CCI) [228, 283]	(i) Antinociceptive [212, 226, 228, 283] (ii) Restores spinal GABA DH inhibition [228] (iii) Colocalize/release GABA and glycine [212]

Human NT2.19 5HT cell line [213, 241, 283]	(i) Contusive SCI [213, 241] (ii) Excitotoxic SCI [283]	(i) Intraspinal grafts attenuate motor dysfunction [213] (ii) Intrathecal grafts provide antinociception [241, 283]
